# Effect of Ultra-Micronized-Palmitoylethanolamide and Acetyl-l-Carnitine on Experimental Model of Inflammatory Pain

**DOI:** 10.3390/ijms22041967

**Published:** 2021-02-17

**Authors:** Alessio Ardizzone, Roberta Fusco, Giovanna Casili, Marika Lanza, Daniela Impellizzeri, Emanuela Esposito, Salvatore Cuzzocrea

**Affiliations:** 1 Department of Chemical, Biological, Pharmaceutical and Environmental Sciences, University of Messina, 98166 Messina (ME), Italy; aleardizzone@unime.it (A.A.); rfusco@unime.it (R.F.); gcasili@unime.it (G.C.); mlanza@unime.it (M.L.); dimpellizzeri@unime.it (D.I.); salvator@unime.it (S.C.); 2 Department of Pharmacological and Physiological Science, Saint Louis University, Saint Louis, MO 63103, USA

**Keywords:** palmitoylethanolamide (PEA), acetyl-l-carnitine (LAC), carrageenan (CAR), edema, inflammatory pain

## Abstract

Palmitoylethanolamide (PEA), a fatty acid amide, has been widely investigated for its analgesic and anti-inflammatory properties. The ultra-micronized formulation of PEA (um-PEA), that has an enhanced rate of dissolution, is extensively used. Acetyl-l-carnitine (LAC), employed for the treatment of neuropathic pain in humans, is able to cause analgesia by up-regulating type-2 metabotropic glutamate (mGlu2) receptors. In the present study, we tested different associations of um-PEA, LAC and non-micronized PEA (non-m-PEA) in a rat model of carrageenan (CAR)-induced paw edema. Intraplantar injection of CAR into the hind paw of animals caused edema, thermal hyperalgesia, accumulation of infiltrating inflammatory cells and augmented myeloperoxidase (MPO) activity. All these parameters were decreased in a significantly manner by oral administration of a compound constituted by a mixture of um-PEA and LAC in relation 1:1 (5 mg/kg), but not with the association of single compounds administered one after the other. These findings showed the superior anti-inflammatory and anti-nociceptive action displayed by oral administration of um-PEA and LAC versus LAC plus, separate but consecutive, um-PEA in the rat paw CAR model of inflammatory pain.

## 1. Introduction

Inflammation and pain are characterized by elevate levels of interleukins and prostaglandins [1]. Current approaches for resolving inflammation consist in targeting ion channels enzymes, epigenetics (for example, histone modification), RNAs (antisense oligonucleotides), and lipid mediators among others. These lipid mediators can act restoring homeostasis and moderate pain sensitivity through the regulation of the flow of nociceptive signals to the central nervous system [2]. Palmitoylethanolamide (PEA), a fatty acid amide which belongs to the family of N-acylethanolamines (NAEs), is considered an endogenous molecule that controls tissue reactivity and the related inflammatory antalgic phenomena [3,4]. It successfully controls neuropathic pain induced by lesions at the peripheral but also at the central nervous system [5,6,7]. The anti-hyperalgesic and anti-inflammatory effects of PEA are explained by several mechanisms: the activation on the cell surface of the cannabinoid (CB) 2-like receptor or the orphan G protein-coupled receptor (GPR)-55 receptor, or the nuclear receptor of the peroxisome proliferator-activated receptors (PPAR) family [8], and the down-regulation of mast cells (MC) degranulation Autacoid Local Inflammation Antagonism (ALIA) mechanism [9]. Moreover, a large body of evidence shows the efficacy of acetyl-l-carnitine (LAC) in the treatment of fibromyalgia, neuropathic, and other types of chronic pain, with a good profile of safety and tolerability [10,11]. It causes analgesia through an epigenetic mechanism intermediated by acetylation of p65/RelA, which is a transcription factor of the nuclear factor kappa-light-chain-enhancer of activated B cells (NF-kB) family. In addition, LAC enhances the production of membrane phospholipids [12] and acts on mitochondrial protein synthesis and non-esterified fatty acid oxidation [13]. Finally, LAC has a free-radical scavenging effect, improving the activity of antioxidant factors and, in this way, protects nerve cells against lipid peroxidation [14]. Carrageenan (CAR)-induced paw edema in rats represents a classical model of inflammation and hyperalgesia [15] that has been widely used in the evaluation of anti-inflammatory drugs. Unfortunately, molecules, such as PEA, have limitations in bioavailability and solubility given their large particle size and lipidic nature. In the pharmaceutical field, the micronization technique is frequently used for dissolution enhancement of poorly water-soluble drugs [16]. This comports a reduced variability of drug absorption when orally administered [17] and enhanced rate of dissolution [18]. Therefore, in the present study, we analyzed the effects of a new formulation containing um-PEA and LAC administered orally in the CAR- induced paw edema, compared to single administrations of non-m-PEA and um-PEA that was administered separately but consecutively with LAC. We aim to demonstrate that um-PEA is able to reduce inflammatory process already at lower dose compared to a higher dose of non-m-PEA. 

## 2. Results

### 2.1. Effect of um-PEA and LAC Treatment on the Time-Course of CAR-Induced Paw Edema and Histological Evaluation of Rat Paw Edema

**Figure 1:**
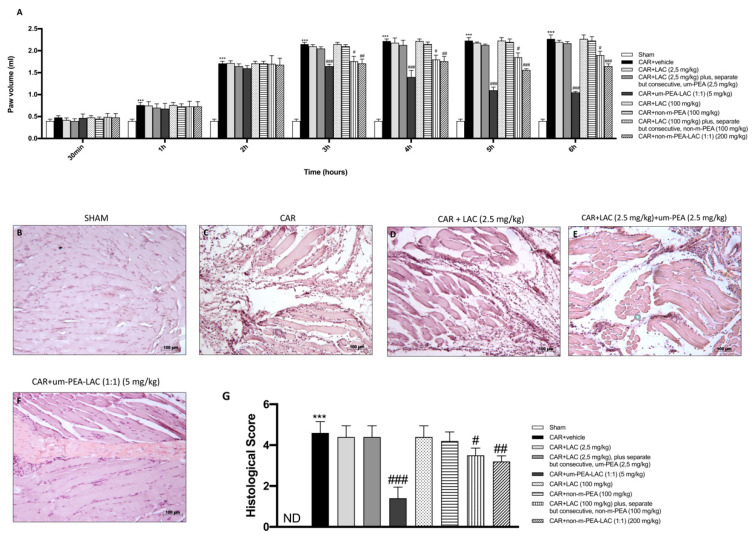
Efficacy of um-PEA and LAC treatment on the time course of CAR-induced paw edema and histological analyses. Um-PEA and LAC (5 mg/kg) significantly reduced CAR-induced paw edema already after 3 h after from CAR injection and thereafter for each time points compared to the CAR-injected group. A less but significant reduction was achieved by treatment with CAR + LAC (100 mg/kg) plus, separate but consecutive, non-m-PEA (100 mg/kg), and CAR + non-m-PEA and LAC groups (1:1) (200 mg/kg). No significant differences were noted in all other groups compared with the vehicle group (**A**). CAR-treated paws showed edema and tissue injury (**C**,**G**) compared to the control (**B**,**G**). Um-PEA and LAC 5 mg/kg treatment significantly reduced tissue injury (**F**,**G**); the reduction in CAR-tissue damage was also appreciable in treatments with LAC (100 mg/kg) plus, separate but consecutive, non-m-PEA (100 mg/kg), and more with non-m-PEA and LAC groups (1:1) (200 mg/kg) (data not shown). Other oral treatments did not show any protection in histological injury induced by CAR injection (**D**,**E**,**G** and data not shown). Data are representative of at least three independent experiments. Values are means ± SEM. One-Way ANOVA test. *** *p* < 0.001 vs. sham; ^#^ *p* < 0.05 vs. CAR; ^##^ *p* < 0.01 vs. CAR; ^###^ *p* < 0.001 vs. CAR. ND not detectable.

Injection of CAR into the hind paw of rats led to a significant time-dependent increase in paw volume (Figure 1A). CAR-induced paw edema was significantly reduced by treatment with um-PEA and LAC (1:1) 5 mg/kg already after 3 h following CAR injection and thereafter for each time points compared to vehicle group (Figure 1A), for each subsequent point until the end of the experimental time. Similar results, but with a milder significance, were also obtained from treatment with CAR + LAC 100 mg/kg plus, separate but consecutive, non-m-PEA (100 mg/kg) (*p* < 0.05). Additionally, the association between non-m-PEA and LAC 200 mg/kg (1:1) was able to reduce paw edema significantly (*p* < 0.01 vs. CAR) in a way that overlaps with PEA and LAC (1:1) (5 mg/kg). Moreover, no significant reduction was observed if um-PEA 2.5 mg/kg was administered separately but consecutively by LAC 2.5 mg/kg (Figure 1A). 

In order to estimate histologically the anti-inflammatory effect of a new association of PEA, paw tissues from each experimental group were observed by Hematoxylin and eosin (H&E) staining. No histologic damage was detected in sham-operated rats (Figure 1B, histological score 1G). In contrast, CAR injection into the hind paw caused a notable accumulation of infiltrating inflammatory cells (Figure 1C, histological score 1G) compared to control group. Inflammatory cell infiltration was meaningfully decreased with um-PEA and LAC (5 mg/kg) treatment (Figure 1F, histological score 1G), conversely, LAC 2.5 mg/kg plus, separate but consecutive, um-PEA 2.5 mg/kg (Figure 1E, histological score 1G) did not reduce the histological scores. Ineffective results were also obtained from treatment with LAC 100 mg/kg (data not shown) and non-m-PEA 100 mg/kg (data not shown).

### 2.2. Effect of um-PEA and LAC Treatment on Myeloperoxidase (MPO) Activity and on the Time-Course of CAR-Induced Thermal Hyperalgesia 

**Figure 2:**
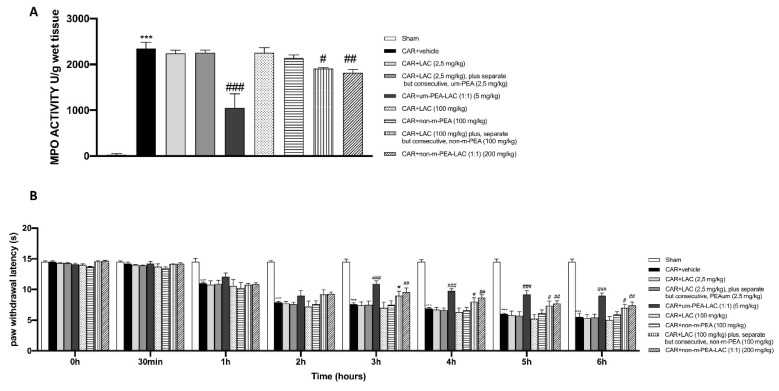
Efficacy of um-PEA and LAC treatment on MPO activity and CAR-induced thermal hyperalgesia. Paw tissues from CAR-treated animals displayed an increased MPO activity compared to the sham group. Um-PEA and LAC (5 mg/kg) treatment produced significant reduction in MPO activity; MPO levels were also discreetly lower in CAR + LAC (100 mg/kg) plus, separate but consecutive, non-m-PEA (100 mg/kg), and CAR + non-m-PEA-LAC groups (1:1) (200 mg/kg). All the other treatments did not show any significant reduction (**A**). Um-PEA and LAC (5 mg/kg) led to an inhibition of the CAR-induced thermal hyperalgesia at 3 h from CAR injection and, also, for subsequent time points, compared to CAR-administrated group. A moderate inhibition was detected in CAR + LAC (100 mg/kg) plus, separate but consecutive, non-m-PEA (100 mg/kg), and CAR + non-m-PEA-LAC groups (1:1) (200 mg/kg). In contrast, all other oral treatments did not attenuate the CAR-induced hyperalgesic response (**B**). Data are representative of at least three independent experiments. Values are means ± SEM. One-Way ANOVA test. *** *p* < 0.001 vs. sham; ^#^ *p* < 0.05 vs. CAR; ^##^ *p* < 0.01 vs. CAR; ^###^ *p* < 0.001 vs. CAR.

The development of histological injury was accompanied by increased infiltration of neutrophils, as revealed by an increase in MPO activity (Figure 2A). The administration um-PEA and LAC 5 mg/kg significantly reduced MPO activity. Conversely, LAC 2.5 mg/kg plus, separate but consecutive, um-PEA 2.5 mg/kg treatment was unable to significantly reduce neutrophil infiltration in the paw tissues from CAR-treated rats, as well as treatments with LAC 100 mg/kg and non-m-PEA 100 mg/kg (Figure 2A). A slight but significant reduction in MPO levels was also found in CAR + LAC (100 mg/kg) plus, separate but consecutive, non-m-PEA (100 mg/kg) and CAR + non-m-PEA-LAC (1:1) (200 mg/kg) groups (Figure 2A).

Intraplantar injection of CAR produced a time-dependent increase in thermal hyperalgesia maintained until 6 h (Figure 2B). Oral administration of um-PEA and LAC (1:1) (5 mg/kg) caused a clear inhibition of the development of CAR-induced thermal hyperalgesia already after 3 h from CAR injection and also for each time points compared to the vehicle group (Figure 2B). An inhibition of the development of CAR-induced thermal hyperalgesia was also detected after LAC (100 mg/kg) plus, separate but consecutive, non-m-PEA (100 mg/kg) and non-m-PEA-LAC (1:1) (200 mg/kg) administrations (Figure 2B).

### 2.3. Effect of um-PEA and LAC Treatment on the Mast Cell Number

**Figure 3:**
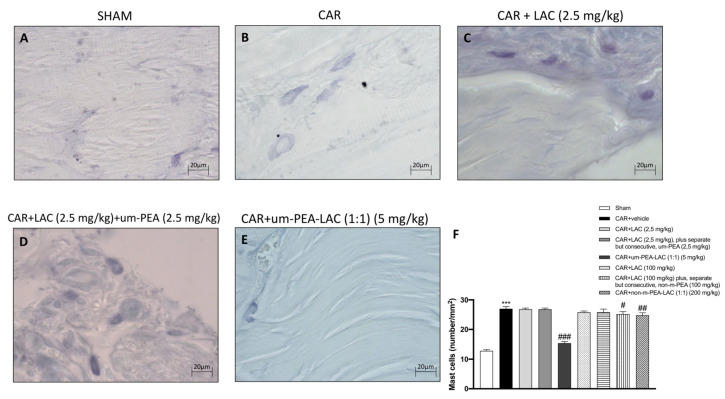
Efficacy of um-PEA and LAC treatment on the mast cells number. An increased number of mast cells was found in tissue from CAR-injected animals (**B**,**F**), compared to the control group (**A**,**F**). A decreased number of mast cells was identified in paw tissues obtained from CAR animals treated with um-PEA and LAC (5 mg/kg) (**E**,**F**) and to a lesser extent in CAR + LAC (100 mg/kg) plus, separate but consecutive, non-m-PEA (100 mg/kg) and CAR + non-m-PEA-LAC groups (1:1) (200 mg/kg) (data not shown). Other remaining treatments did not show any significant reduction (**C**,**D**,**F,** and data not shown). Data are representative of at least three independent experiments. Values are means ± SEM. One-Way ANOVA test. *** *p* < 0.001 vs. sham; ^#^ *p* < 0.05 vs. CAR; ^##^ *p* < 0.01 vs. CAR; ^###^ *p* < 0.001 vs. CAR.

Mast cells and histamine have a central role in edematogenic activity [19,20]. Toluidine blue staining evidenced the presence of mast cells in paw tissues 6 h after edema induction (Figure 3F). Tissue collected from CAR-injected animals displayed an increased number in mast cells (Figure 3B,F), compared to the sham-treated group (Figure 3A,F). In contrast, a lower number of mast cells was detected in paw tissues from CAR animals treated with um-PEA and LAC (5 mg/kg) (Figure 3E,F) compared to the vehicle group (Figure 3B,F). Treatment with LAC 2.5 mg/kg plus, separate but consecutive, um-PEA 2.5 mg/kg (Figure 3D,F) did not reduce the number of mast cells induced by CAR administration. A considerable decrease (although not as powerful as um-PEA and LAC 5 mg/kg) was achieved by treatment with CAR + non-m-PEA and LAC (1:1) (200 mg/kg) (data not shown).

### 2.4. Effect of um-PEA and LAC Treatment on Intercellular Adhesion Molecule 1 (ICAM-1) Expression 

**Figure 4:**
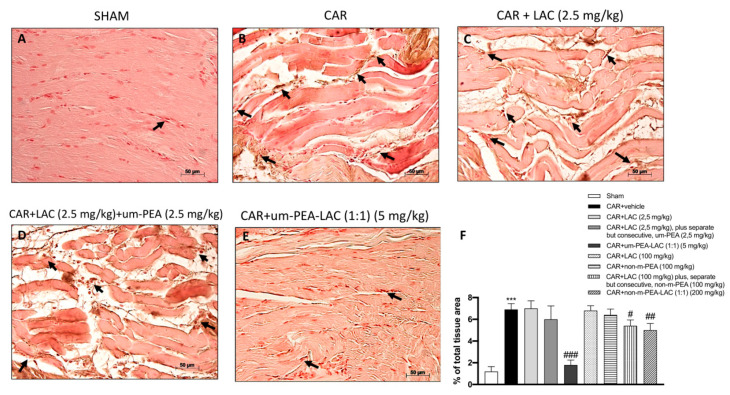
Efficacy of um-PEA and LAC treatment on ICAM-1 expression. Paw tissues collected from CAR treated-animals showed positive immunostaining for ICAM-1 (**B**,**F**), compared to the sham group (**A**,**F**). CAR + LAC (100 mg/kg) plus, separate but consecutive, non-m-PEA (100 mg/kg) and CAR + non-m-PEA and LAC groups (1:1) (200 mg/kg) slightly reduced positive immunostaining (data not shown); while um-PEA and LAC (5 mg/kg) administration strongly reduced this expression (**E**,**F**). Other administrations, individually or mixed, did not show a significant down-regulation of ICAM-1 expression (**C**,**D**,**F,** and data not shown). Black arrows indicate the positive staining for ICAM-1. Data are representative of at least three independent experiments. Values are means ± SEM. One-Way ANOVA test. *** *p* < 0.001 vs. sham; ^#^ *p* < 0.05 vs. CAR; ^##^ *p* < 0.01 vs. CAR; ^###^ *p* < 0.001 vs. CAR.

Immunohistochemical analysis for ICAM-1 displayed a constitutive expression of this adhesion molecule in paw tissue sections obtained from sham-treated rats (Figure 4A,F). After 6 h from CAR injection, a substantial increase in ICAM-1 staining along the paw tissue vessels was detected in CAR-treated rats (Figure 4B,F). Vice versa, ICAM-1 staining was significantly reduced in paw tissue collected from CAR-treated rats and those subjected to treatment with um-PEA and LAC (5 mg/kg) (Figure 4E,F); this reduction was lower but still appreciable in CAR + non-m-PEA and LAC (1:1) (200 mg/kg) groups (data not shown).

### 2.5. Effect of um-PEA and LAC Treatment on Tumor Necrosis Factor-α (TNF-α) Expression 

**Figure 5:**
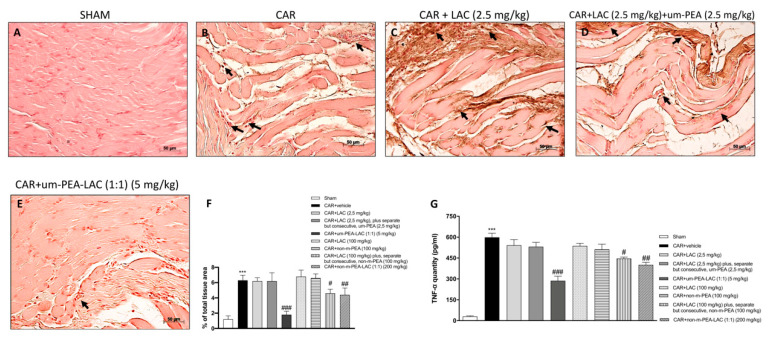
Efficacy of um-PEA and LAC treatment on TNF-α expression. Positive TNF-α immunostaining was found in paw tissues collected from vehicle treated rats (**B**,**F**), compared to the sham-treated rats (**A**,**F**). CAR + LAC (100 mg/kg) plus, separate but consecutive, non-m-PEA (100 mg/kg) and CAR + non-m-PEA and LAC (1:1) (200 mg/kg) treatments (data not shown), but even more um-PEA and LAC (5 mg/kg) administration reduced this staining (**E**,**F**). Other treatments, individually or mixed, did not show any significant reduction in TNF-α expression (**C**,**D**,**F** and data not shown). Black arrows indicate the positive staining for TNF-α. Quantification of TNF-α levels confirmed data (**G**). Data are representative of at least three independent experiments. Values are means ± SEM. One-Way ANOVA test. *** *p* < 0.001 vs. sham; ^#^ *p* < 0.05 vs. CAR; ^##^ *p* < 0.01 vs. CAR; ^###^ *p* < 0.001 vs. CAR.

Immunohistochemical analysis of TNF-α showed a significant increased expression in paw tissue from CAR injected rats (Figure 5B,F), whereas um-PEA and LAC (5 mg/kg) reduced such positive staining (Figure 5E,F). Such positive immunostaining was also significantly reduced by treatment with CAR + non-m-PEA and LAC (1:1) (200 mg/kg) groups (data not shown). Treatment with LAC 2.5 mg/kg plus, separate but consecutive, um-PEA 2.5 mg/kg (Figure 5D,F) did not attenuate the CAR-induced TNF-α expression, compared to the sham-treated group (Figure 5A,F). In all other experimental groups, no significant reductions were found (data not shown).

The data obtained from the immunohistochemistry analysis of TNF-α were confirmed by the quantitative ELISA assay as shown in Figure 5G.

### 2.6. Effect of um-PEA and LAC Treatment on IL-1β Expression 

**Figure 6:**
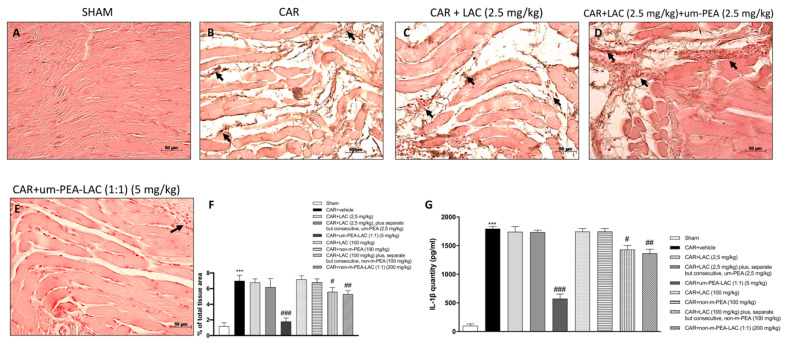
Efficacy of um-PEA and LAC treatment on IL-1β expression. Tissues from CAR injected-animals displayed positive immunostaining for IL-1β (**B**,**F**), compared to the sham group (**A**,**F**). Um-PEA and LAC (5 mg/kg) administration decreased this expression (**E**,**F**); but also CAR + LAC (100 mg/kg) plus, separate but consecutive, non-m-PEA (100 mg/kg) and CAR + non-m-PEA and LAC (1:1) (200 mg/kg) treatments (data not shown), albeit less effectively. Other administrations, individually or mixed, did not show a significant difference in IL-1β expression with the CAR injected-group (**C**,**D**,**F,** and data not shown). Black arrows indicate the positive staining for IL-1β. Quantification of IL-1β levels confirmed data (**G**). Data are representative of at least three independent experiments. Values are means ± SEM. One-Way ANOVA test. *** *p* < 0.001 vs. sham; ^#^ *p* < 0.05 vs. CAR; ^##^ *p* < 0.01 vs. CAR; ^###^ *p* < 0.001 vs. CAR.

After six hours from CAR injection, immunohistochemical analysis for IL-1β expression was performed. IL-1β expression was increased in paw tissue collected from CAR treated rats (Figure 6B,F), while um-PEA and LAC (5 mg/kg) significantly reduced the CAR-induced IL-1β expression (Figure 6E,F). An albeit less significant reduction in IL-1β expression was also found in the CAR + non-m-PEA-LAC groups (1:1) (200 mg/kg). Administration of LAC 2.5 mg/kg (Figure 6C,F) and LAC 2.5 mg/kg plus, separate but consecutive, m-PEA 2.5 mg/kg (Figure 6D,F) did not reduce the CAR-induced IL-1β expression, compared to the sham group (Figure 6A,F). None of the other treatments had the ability to reduce the expression of IL-1β (data not shown).

In order to verify the results obtained from the immunohistochemistry analysis of IL-1β we performed a quantitative assay by ELISA kit, confirming the data (Figure 6G).

### 2.7. Effect of um-PEA and LAC Treatment on Cyclooxygenase-2 (COX-2) Expression 

**Figure 7:**
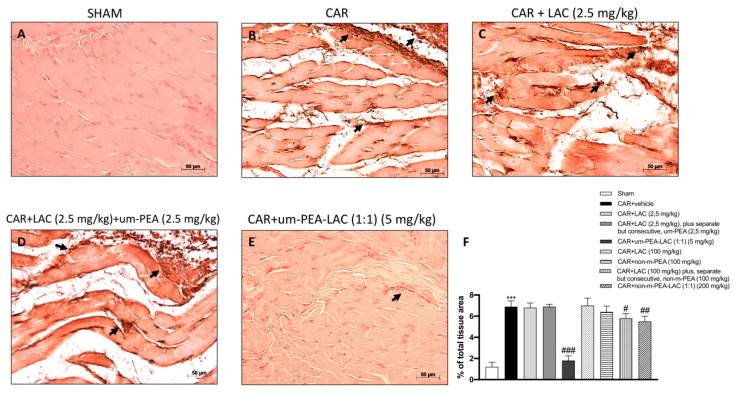
Efficacy of um-PEA and LAC treatment on COX-2 expression. Immunohistochemical analysis of COX-2 in the paw tissue from sham-treated rats (**A**,**F**) or from CAR-injected animals (**B**,**F**) are shown. The intensity of the COX-2 positive staining was markedly reduced in tissue section obtained from CAR-injected animals which have been treated with um-PEA and LAC (5 mg/kg) (**E**,**F**), and in a reduced way with CAR + LAC (100 mg/kg) plus, separate but consecutive, non-m-PEA (100 mg/kg) and CAR + non-m-PEA and LAC (1:1) (200 mg/kg) (data not shown). While other treatments did not show any down-regulation of COX-2 expression induced by CAR-injection (**C**,**D**,**F** and data not shown). Black arrows indicate the positive staining for COX-2. Data are representative of at least three independent experiments. Values are means ± SEM. One-Way ANOVA test. *** *p* < 0.001 vs. sham; ^#^ *p* < 0.05 vs. CAR; ^##^ *p* < 0.01 vs. CAR; ^###^ *p* < 0.001 vs. CAR.

After six hours, post-carrageenan injection, positive expression of COX-2 was found in tissues from CAR administrated animals (Figure 7B,F) compared to the control group (Figure 7A,F). Um-PEA and LAC (5 mg/kg) administration significantly reduced the COX-2 expression (Figure 7E,F), while LAC 2.5 mg/kg plus, separate but consecutive, um-PEA 2.5 mg/kg treatments did not show a significant reduction on COX-2 expression induced by CAR injection (Figure 7D,F). CAR + LAC (100 mg/kg) plus, separate but consecutive, non-m-PEA (100 mg/kg) and CAR + non-m-PEA and LAC (1:1) (200 mg/kg) groups were able to slightly moderate COX-2 expression (data not shown). COX-2 expression was not remarkably reduced by the other treatments (data not shown).

### 2.8. Effect of um-PEA and LAC Treatment on Inducible Nitric Oxide Synthase (iNOS) Expression 

**Figure 8:**
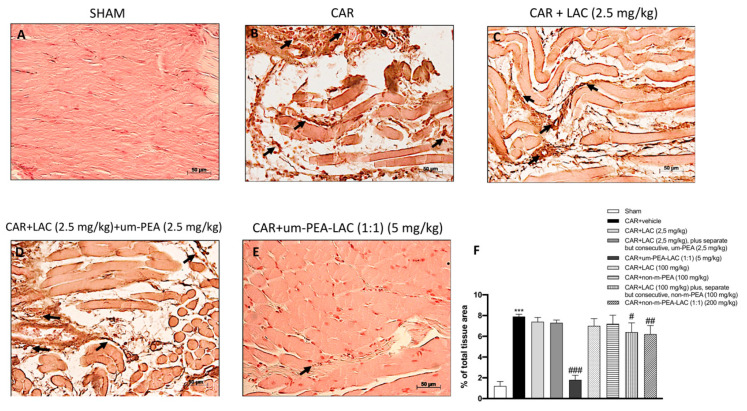
Efficacy of um-PEA and LAC treatment on iNOS expression. Tissues obtained from CAR injected-animals exhibited positive immunostaining for iNOS (**B**,**F**), compared to the sham-treated animals (**A**,**F**). Um-PEA and LAC (5 mg/kg) treatment reduced this expression (**E**,**F**); this reduction was milder with CAR + LAC (100 mg/kg) plus, separate but consecutive, non-m-PEA (100 mg/kg) and CAR + non-m-PEA and LAC (1:1) (200 mg/kg) (data not shown). Other treatments did not display a significant decrease in iNOS expression (**C**,**D**,**F,** and data not shown). Black arrows indicate the positive staining for iNOS. Data are representative of at least three independent experiments. Values are means ± SEM. One-Way ANOVA test. *** *p* < 0.001 vs. sham; ^#^ *p* < 0.05 vs. CAR; ^##^ *p* < 0.01 vs. CAR; ^###^ *p* < 0.001 vs. CAR.

The effect of all treatments was studied on the pro-inflammatory enzyme iNOS after CAR injection. The expression of iNOS was upregulated in paw tissues after CAR injection (Figure 8B,F) compared to the sham group (Figure 8A,F). Treatment with um-PEA and LAC (5 mg/kg) was able to strongly reduce iNOS expression induced by CAR injection (Figure 8E,F); while a smaller but still significant reduction was detected in the paws of CAR + LAC (100 mg/kg) plus, separate but consecutive, non-m-PEA (100 mg/kg) and CAR + non-m-PEA and LAC (1:1) (200 mg/kg) rats (data not shown). All other experimental groups did not reduce meaningfully iNOS immunostaining (8C, 8D, 8F, and data not shown).

## 3. Discussion

In this study, we investigated a new pharmacological approach for controlling the development of paw edema involved in the acute inflammatory process. We analyzed the effect of different formulations of um-PEA, LAC, and non-m-PEA which seems to have protective and anti-nociceptive effect in rats receiving an intraplantar injection of CAR. In particular, here we showed that oral administration of LAC at 2.5 mg/kg and also LAC 2.5 mg/kg plus, separate but consecutive, um-PEA 2.5 mg/kg did not have any significant effect in modulating the acute inflammatory process caused by CAR-induced paw edema. This ineffectiveness in reducing inflammatory condition was maintained even at much higher doses of both LAC and PEA; in fact, single administrations of LAC (100 mg/kg) or PEA (100 mg/kg) in its non-micronized form have shown unsuccessful results in counteracting CAR-inflammation if administered consecutively and separately. On the contrary, a single administration of the compound constituted by um-PEA and LAC 5 mg/kg was able to regulate inflammation and nociception in a rat model of CAR-induced paw edema, proving to be the most effective treatment, even more so than treatment with non-m-PEA-LAC (1:1) (200 mg/kg), which despite being fairly effective in moderating CAR-damage inflammation but did not reach the effectiveness of um-PEA and LAC (5 mg/kg) co-administration. Injection of CAR into the paw tissue resulted in tissue damage, thermal hyperalgesia, and infiltration of inflammatory cells (MPO activity) [21]. Mast cells also play a central role in the development of paw edema, they are multifactorial immune cells that enclose many inflammatory mediators [22]. The inflammatory response to CAR-induced edema led to an increased number of mast cells [23] and a release of serotonin and histamine [24]. Conceivably, the reduction in CAR-induced nociception was due to mast cells modulation by um-PEA and LAC co-administration, but not to the administration of LAC 2.5 mg/kg plus, separate but consecutive, um-PEA 2.5 mg/kg or other administrations. Increasing evidences suggest the crucial role of ICAM-1 in the pathological process associated with the development of edema [25]. Because treatment with um-PEA and LAC reduced ICAM-1 up-regulation, it could also reduce the interaction between neutrophils and endothelial cells facilitated by ICAM-1. These results are well in line with the reduced leukocyte migration in paw tissues indicated by MPO. Um-PEA and LAC treatment strongly decreased MPO paw tissues, compared to LAC 2.5 mg/kg plus, separate but consecutive, um-PEA 2.5 mg/kg treated rats. Early stages of inflammation usually are characterized by an up-regulation of cytokines (such as TNF-α and IL-1β) and pro-inflammatory enzymes (such as COX-2 and iNOS) expression [25]. In this study, we showed that um-PEA and LAC treatment was able to powerfully reduce cytokines and pro-inflammatory enzymes levels during paw inflammation, proving to be the most effective treatment compared to other single or mixed treatments at various dosages, which did not exhibit this capability. 

## 4. Materials and Methods 

### 4.1. Materials

Unless otherwise stated, all compounds employed in this study were obtained from Sigma–Aldrich (Poole, UK). LAC, um-PEA, non-m-PEA, and various mixtures, as well as carboxymethylcellulose (CMC), were obtained from Epitech Group Spa (Saccolongo, Italy). 

The difference between non-m-PEA and um-PEA is based on the particle size of the molecule. Given their lipidic nature and large particle size in the native state, molecules such as PEA may have limitations in terms of solubility and bioavailability. The use of micronization for dissolution enhancement of poorly water-soluble drugs is a technique frequently used in the pharmaceutical field. By application of this technique, microparticles are produced by reducing large drug crystals down to the micron range (<10 μm). Given that the dissolution rate of a drug is proportional to its surface area, major benefits of microcrystal formulations are enhanced rate of dissolution and reduced variability of drug absorption when orally administered. The um-PEA has and average particle size of 0.8 ± 2 microns; this size makes able PEA to cross both the intestinal mucosa and the blood brain barrier (BBB) reaching an effective concentration [16].

All solutions used for in vivo infusion were prepared using a nonpyrogenic saline solution (0.9% *w/v* NaCl; Baxter Healthcare, Thetford, UK). 

### 4.2. Animals

The study was performed using male Sprague–Dawley rats (200–235 g; Harlan, Nossan, Italy). Food and water were accessible ad libitum. The study was permitted by the University of Messina Review Board for the animals’ care and regulated with Italian straight on the protection of animals employed for experimental and scientific purposes (DM116192), as well as with the European Economic Community (EEC) guidelines (OJ of EC L 358/1 12/18/1986).

### 4.3. CAR-Induced Paw Edema

Rats were concurrently subjected to a singular subplantar injection of CAR (100 µL of saline solution containing 1% CAR). Paw edema was measured with a plethysmometer (Ugo Basile, Comerio, Varese, Italy) [26] prior to CAR injection and every hour for 6 h. Edema was expressed as the increase in paw volume (mL) after CAR injection relative to the pre-injection value for all animals. Scores were expressed as paw volume difference (mL). 

### 4.4. Experimental Groups

Rats were randomly divided into the following groups:

Group 1: The Sham-operated group received the same surgical procedures of the CAR group, excepting that saline solution was administered instead of CAR (*N* = 10).

Group 2: CAR + vehicle group: rats were subjected to CAR-induced paw edema and received orally by gavage the vehicle CMC (1.5% *w/v* in saline solution) 30 min before CAR (*N* = 10);

Group 3: CAR + LAC 2.5 mg/kg: rats were subjected to CAR-induced paw edema and received orally by gavage LAC 2,5 mg/kg dissolved in CMC (1.5% *w/v* in saline solution) 30 min before CAR (*N* = 10);

Group 4: CAR + LAC 2.5 mg/kg plus, separate but consecutive, um-PEA 2.5 mg/kg: rats were subjected to CAR-induced paw edema and received orally by gavage LAC 2.5 mg/kg plus, separate but consecutive, um-PEA 2.5 mg/kg dissolved CMC (1.5% *w/v* in saline solution) 30 min before CAR (*N* = 10);

Group 5: CAR + um-PEA/LAC (1:1) (5 mg/kg): rats were subjected to CAR-induced paw edema and received orally by gavage um-PEA/LAC mixture in 1:1 ratio (5 mg/kg) dissolved in CMC (1.5% *w/v* in saline solution) 30 min before CAR (*N* = 10);

Group 6: CAR + LAC 100 mg/kg: rats were subjected to CAR-induced paw edema and received orally by gavage LAC 100 mg/kg dissolved in CMC (1.5% *w/v* in saline solution) 30 min before CAR (*N* = 10);

Group 7: CAR + non-m-PEA 100 mg/kg: rats were subjected to CAR-induced paw edema and received orally by gavage non-m-PEA 100 mg/kg (*N* = 10);

Group 8: CAR + LAC 100 mg/kg plus, separate but consecutive, non-m-PEA 100 mg/kg: rats were subjected to CAR-induced paw edema and received orally by gavage LAC 100 mg/kg and separate but consecutive non-m-PEA 100 mg/kg dissolved in CMC (1.5% *w/v* in saline solution), 30 min before CAR (*N* = 10);

Group 9: CAR + non-m-PEA and LAC (1:1) 200 mg/kg: rats were subjected to CAR-induced paw edema and received orally by gavage non-m-PEA and LAC 200 mg/kg mixture in 1:1 ratio dissolved in CMC (1.5% *w/v* in saline solution) 30 min before CAR (*N* = 10);

### 4.5. Nociceptive Tests

Hyperalgesic responses to heat were assessed using the Plantar Test (Hargreaves method, Ugo Basile, Comerio, Varese, Italy) with a cut-off latency of 20 s to avoid tissue damage [27]. Rats were individually housed in Plexiglas compartments and allowed to habituate. A mobile unit consisting of a high-intensity projector bulb was positioned to deliver a thermal stimulus directly to an individual hind paw from beneath the chamber. The withdrawal latency period of injected paws was determined with an electronic clock circuit and thermocouple. Results were expressed as paw withdrawal latencies changes (s). Behavioral testing was done with the experimenter blinded to treatment conditions. 

### 4.6. Histological Evaluation

Paw tissues were collected 6 h after CAR injection. Samples were fixed in 10% formaldehyde solution in phosphate buffer saline (PBS) at room temperature for 24 h, dehydrated by graded series of ethanol and at the list embedded in Paraplast (Sherwood Medical, Mahwah, NJ, USA). Sections of 7-μm thickness were cut with a microtome, deparaffinized with xylene and stained with hematoxylin and eosin (H&E). All sections were examined using an Axiovision Zeiss (Milan, Italy) microscope by two investigators blinded to the treatment conditions. The histological analysis was performed according to a previously described method [27] and given the following score from 0 to 5: 0 = no inflammation, 1 = mild inflammation, 2 = mild/moderate inflammation, 3 = moderate inflammation, 4 = moderate/severe inflammation and 5 = severe inflammation.

### 4.7. Myeloperoxidase Activity

The activity of MPO (an enzyme released by neutrophils and used as a marker of neutrophil infiltration) was assessed as previously described [28]. The rate of change in absorbance was calculated spectrophotometrically at 650 nm. MPO activity was measured in U per gram weight of wet tissue and was quantified as the quantity of enzyme degrading 1μmol of peroxide 1 min at 37 °C.

### 4.8. Toluidine Blue Staining

In order to evaluate mast cells number in the paw sections, tissues were stained with toluidine blue. Sections were deparaffinized in xylene and next dehydrated through a graded series of ethanol, for 5 min in each solution. The sections were then placed in water for 5 min, stained with toluidine blue for 4 min and then blotted carefully. Sections were located in absolute alcohol for 1 min, cleared in xylene, and mounted on a glass slide using Eukitt (Bio-Optica, Italy, Milan). Sections were stained blue and the mast cells were stained purple. The mast cells count was carried out on each slide using an Axiovision Zeiss (Milan, Italy) microscope. For toluidine blue staining images 40× (20 µm scale bar) were shown.

### 4.9. Immunohistochemical Localization of ICAM-1, TNF-α, IL-1β, COX-2, and iNOS

After six hours, post the induction of CAR edema, the animals were sacrificed and tissue collected for immunohistochemical analysis. Paw samples were fixed in formaldehyde (10% PBS-buffered) and next embedded in paraffin. Samples were cut with microtome into 7 µm slices and, after deparaffinization, endogenous peroxidase was quenched with 0.3% (*v/v*) hydrogen peroxide in 60% (*v/v*) methanol for 30 min. Slides were permeabilized with 0.1% (*w/v*) Triton X-100 in PBS for 20 min. Non-specific adsorption was decreased by incubating the section in 2% (*v/v*) normal goat serum in PBS for 20 min. Endogenous avidin or biotin binding sites were blocked by sequential incubation for 15 min with avidin and biotin (Vector Laboratories, Burlingame, CA, USA), respectively. Then sections were immunostained overnight with the following antibodies: polyclonal rabbit anti-ICAM-1 (sc-8439, Santa Cruz Biotechnology, CA, USA):), monoclonal mouse anti-TNF-α (sc-52746, Santa Cruz Biotechnology, CA, USA), monoclonal mouse anti-IL-1β (sc-32294, Santa Cruz Biotechnology, CA, USA), monoclonal mouse anti-COX-2 (sc-376861, Santa Cruz Biotechnology, CA, USA) and monoclonal mouse anti-iNOS (610432 BD Transduction, CA, USA). Sections were washed with PBS and incubated with secondary antibody. Specific labelling was detected with a biotin-conjugated goat anti-rabbit IgG and avidin–biotin peroxidase complex (Vector Laboratories, Burlingame, CA, USA). Immunohistochemistry photographs were acquired with Leica DM2000 microscope and analyzed by densitometry using Optilab Graftek software. For immunohistochemistry images, 20× (50 µm scale bar) were shown.

### 4.10. ELISA Assay for TNF-α and IL-1β

TNF-α and IL-1β levels were evaluated in the supernatants of the rats of each experimental group, as previously performed and described by Casili. et al. [29].

Cytokines levels were measured by ELISA kit, according to the manufacturer's instructions (Thermo Fisher Scientific, Waltham, MA, USA), through colorimetric microplate reader.

### 4.11. Statistical Analysis

All values are expressed as mean ± standard error of the mean (SEM) of N observations. For the in vivo studies, N represents the number of animals studied. 

In the experiments involving histology or immunohistochemistry, the figures shown are representative of at least three experiments, performed on different experimental days, on the tissue sections collected from all the animals in each group. Data were analyzed by one-way ANOVA followed by a Bonferroni post-hoc test for multiple comparisons. Non-parametric data were analyzed with the Fisher's exact test. A *p*-value less than 0.05 will be considered significant. 

## 5. Conclusions

In this study, we demonstrate that um-PEA and LAC exerts anti-nociceptive and anti-inflammatory effects by inhibiting the production of inflammatory associated mediators. These data encourage future testing of the um-PEA and LAC, which in addition to having a better absorption thanks to its ultramicronized form, has shown a better synergy thanks to its co-administration instead of different but consequently administration of the two compounds.

## 6. Patents

Salvatore Cuzzocrea is co-inventor on patent WO2013121449 A8 (Epitech Group SpA) which deals with compositions and methods for the modulation of amidases capable of hydrolysing Nacylethanolamines useable in the therapy of inflammatory diseases. Moreover, Dr. Cuzzocrea is also a co-inventor with Epitech group on the following patents: 1. EP 2 821 083 2. MI2014 A001495 3. 102015000067344.

## Data Availability

The data presented in this study are available on request from the corresponding author.

## Abbreviations

PEA	Palmitoylethanolamide
PEA-um or um-PEA	Ultra-micronized-palmitoylethanolamide
non-m-PEA	PEA non-micronized
LAC	Acetyl-l-carnitine
CAR	Carrageenan
PPARs	Peroxisome proliferator-activated receptors
NAEs	N-acylethanolamines
MC	Mast cells
PALS	Phase analysis light scattering
mGlu2	Type-2 metabotropic glutamate receptors
